# Measuring chronic stress exposure incorporating the active and healthy ageing (AHA) concept within the cross-sectional Bern cohort study 2014 (BeCS-14)

**DOI:** 10.1186/s13030-019-0143-6

**Published:** 2019-02-12

**Authors:** Petra Stute, Marion Anker, Laura Hollenstein, Michael von Wolff, Norman Bitterlich, Florian Meissner, Dagmar Poethig

**Affiliations:** 1 Department of Gynecologic Endocrinology and Reproductive Medicine, University Clinic of Obstetrics and Gynecology, Inselspital Bern, Effingerstrasse 102, 3010 Bern, Switzerland; 2 Departement of Visceral and General Surgery, Spital Thun STS, Thun, AG Switzerland; 3 Departement of Internal Medicine, Spital Oberengadin, Samedan, Switzerland; 4 Medizin & Service GmbH, Chemnitz, Germany; 5 vital.services GmbH, Im GerontoLabEurope, Leipzig, Germany; 6 European Association on Vitality and Active Aging eVAA e.V, Leipzig, Germany

**Keywords:** Chronic stress, European innovation partnership on active and healthy ageing (EIP-AHA), Bio-functional status, Bio-functional age, Bern cohort study 2014

## Abstract

The aim of the study was to represent chronic stress exposure by a complex generic Active and Healthy (AHA) diagnostic assessment tool incorporating ICF. This is a single-centre, cross-sectional, observational, non-interventional, non-randomized trial in University based women’s hospital, division of Gynecological Endocrinology and Reproductive Medicine. All participants followed a standardized, holistic battery of biopsychosocial assessments consisting of bio-functional status (BFS), bio-functional age (BFA) and the questionnaire for chronic stress exposure (TICS). 624 non-pediatric, non-geriatric subjects were recruited in the BeCS-14 cohort. The mean difference between chronological age and BFA was 7.8 ± 8.0 year equivalents. The mean stress level score assessed by SSCS was 13.2 with 45.4% being exposed to above average stress intensity. 22 BFS items (14 objective, 7 subjective) significantly correlated with chronic stress exposure (TICS-SSCS). The constructed sum score composed of SOC_L9 and complaint questionnaire (physical and emotional wellbeing) represented chronic stress exposure best (pearson-correlation value 0.564, *p* < 0.0001). Higher chronic stress exposure was associated with bio-functional pro-aging (less vitality) in both sexes. In conclusion, chronic stress is accepted as a major risk factor for developing non-communicable diseases (NCD). Our ICF compatible, complex, generic BFS/BFA assessment tool reflects chronic stress exposure and may be applied in various health care settings, e.g., in health promotion and prevention of NCDs.

## Introduction

The concept of Active and Healthy Aging (AHA) incorporates biological aging, active aging as well as changes in psychological and social wellbeing [1] that may support survival to old age, delay in the onset of non-communicable diseases (NCD) and optimal functioning for the maximal period at individual levels, body systems and cells. The corresponding conceptual AHA framework [1] includes several items such as functioning (individual capability and underlying body systems), wellbeing, activities and participation, and diseases including NCD.

Chronic stress defined as maladaption to repeatedly occuring stressors due to the absence of adaequate coping mechanisms [2] has an impact on AHA as chronic activation of stress regulating systems, e.g. the sympathetic nervous system and the hypothalamic-pituitary-adrenal (HPA) axis, are associated with an increased risk for several NCD such as cardiovascular diseases [3], gastrointestinal diseases [4], diabetes mellitus, osteoporosis, immunodeficiency [5], sleeping disorders [6] and chronic pain [7], respectively.

We recently fitted our bio-functional status (BFS) and bio-functional age (BFA) assessment tool into a theoretical model incorporating both, the ICF and AHA concept, resulting in a complex generic AHA assessment diagnostic tool [8]. This complex generic AHA assessment diagnostic tool meets the EIP-AHA requirements for a diagnostic AHA instrument [9] such as applicability to health and disease across age stages (non-pediatric and non-geriatric lifetime), easy, partly self- and proxy administration, and accordance with the ICF of the WHO. As chronic stress is one major factor influencing AHA we postulate that our complex generic AHA assessment diagnostic tool also reflects chronic stress exposure. To test this assumption, we applied the BFS/BFA assessment tool together with the validated questionnaire for chronic stress exposure, TICS, within our Bern Cohort Study 2014 (BeCS-14) and aimed to generate a score from selected BFS items to represent chronic stress exposure not only subjectively [10] but also objectively.

## Material and methods

### Study population

German speaking women and men aged 18 to 65 were recruited between 04.03.2012 and 04.07.2014 at the Department of Obstetrics and Gynecology, Inselspital Bern, Switzerland. Recruitment was performed by the principle investigator (PS), the study nurse (JDW) and fourteen doctoral students of the medical school, university Bern, via personal contact (patients, collegues, family, friends) and online advertisement (internet, intranet Inselspital Bern, social media). Exclusion criteria were pregnancy, acute diseases (e.g. fever, acute pain syndrome), and illiteracy. The study protocol was approved by the cantonal ethics committee Bern (ref.-Nr. KEK-BE: 023112), and written informed consent was obtained from each participant

### Study design

The study design has been described before [8]. Briefly, this was a single-centre, cross-sectional, observational, non-interventional trial. All participants within BeCS-14 followed a standardized battery of assessments consisting of a personal and family history, bio-functional status (BFS) and bio-functional age (BFA), and validated questionnaires for depression and anxiety (HADS) [11], health-related quality of life (SF-36) [12] and chronic stress (TICS) [13], respectively. Participants were further divided into four subgroups for additional assessments addressing “nutrition” by AD-EVA [14] and PATEF [15], “employees’ health” by IMPULS [16], “stress” by heart rate variability [17] and “cognition” by IGD [18]. The assessments relevant for this publication are further described in section 2.3.

### Assessment procedures

#### Personal and family history

Briefly, we assessed age, social status (partnership, having children, satisfaction with relationship and sex life), life style (alcohol, tobacco, sport, sleep), and job status (highest educational degree, current field of work, job position, working hours, monthly gross income, presenteeism, absenteeism). Personal and family history further comprised information about malignancy, cardiovascular disease, breathing disorder, abdominal and urogenital disease, metabolic disorder, skin and/or hair disease, neuromuscular and psychiatric disorder as well as bone and joint disease.

#### Bio-functional status (BFS) and bio-functional age (BFA)

The BFS was assessed by a comprehensive test battery developed by Poethig et al. and reported by others [19–21], respectively, which is commercially available via vital.services (https://www.microsoft.com/de-de/p/biofunktionaler-status/9wzdncrd38qr). The test battery comprises holistic characteristics from physical, mental-emotional and social areas that fit into a complex theoretical model incorporating the ICF and AHA concept (Table 1). The test battery for BFS assessment is a validated age- and sex-specific tool (objectivity 0.96, reliability 0.93, females age validity: total age correlation 85.2%; total age commonality in the main factor 76.3%). The BFA is based on a sex-specific regression and factor analysis of functional age [19–22].

**Table 1:** Single items of the bio-functional status (BFS) and calculated bio-functional age (BFA)

Bio-functional status (BFS) item	N	Mean	SD	5th–95th percentile	Min - Max
Physical parameters					
Systolic blood pressure [mmHg]	612	118.9	13.8	99.0–144.0	82.0–169.0
Diastolic blood pressure [mmHg]	612	74.4	10.6	58.7–93.0	47.0–107.0
Resting heart rate (p0) [n/min]	607	68.3	10.4	52.4–86.0	42.0–107.0
Pulse rate difference (Δp)	600	121.1	18.4	92.0–152.0	68.0–204.0
Performance time [sec]	600	24.0	6.0	16.6–34.4	12.1–63.0
Pulse performance index (PPI) [Δp/performance time]	600	2.3	0.1	1.0–4.1	0.1–8.3
Vital capacity [l]	592	4.2	1.2	2.5–6.7	1.7–7.5
Vital capacity, women [%]	441	106.8	18.9	73.5–136.6	43.0–158.7
Vital capacity, men [%]	150	110.8	18.1	78.9–137.1	54.5–153.0
Hand grip strength, left [KP]	598	33.2	18.0	9.0–70.0	1.0–70.0
Hand grip strength, right [KP]	602	38.4	18.1	14.0–70.0	1.0–90.0
Hand grip strength, both sides [KP]	600	71.5	35.1	22.0–140.0	2.0–140.0
Body cell mass (%)	587	40.8	4.6	32.9–48.1	28.1–55.0
Body cell mass – active cell mass [kg]	587	27.6	5.0	22.0–37.7	19.5–48.5
Body cell mass – body water [kg]	587	35.1	6.4	28.0–47.9	24.8–61.8
Body cell mass – lean body mass [kg]	587	47.9	8.7	38.2–65.4	33.8–84.5
Fat mass (%)	587	29.3	8.0	16.4–42.9	4.8–51.4
Fat mass [kg]	587	20.5	8.4	10.3–36.1	1.9–56.5
BIA RC 50 (Ohm)	587	608.7	85.8	465.0–744.0	368.0–869.0
BIA XC 50 (Ohm)	587	63.2	10.8	47.0–80.0	30.0–116.0
Teeth status - decayed, missing or filled teeth (n)	616	9.9	8.1	0.0–28.0	0.0–32.0
Body weight (kg)	617	68.3	13.1	51.0–94.0	36.0–120.0
Body height (cm)	617	169.8	8.7	157.0–185.0	150.0–195.0
Body mass index [kg/m2]	617	23.7	4.0	18.7–31.2	12.3–47.0
Sensory physiology and psychomotor parameters					
Vision left [%]	368	81.2	30.1	10.0–100.0	0.0–100.0
Vision right [%]	367	81.0	29.2	12.0–100.0	0.0–100.0
Hearing acuity -HV1024 left (Db)	551	16.0	8.7	5.0–30.4	0.0–100.0
Hearing acuity -HV1024 left (%)	551	1.6	2.5	0.0–5.40	0.0–30.0
Hearing acuity -HV1024 right (Db)	551	17.5	8.6	7.0–30.0	0.0–77.0
Hearing acuity -HV1024 right (%)	551	2.0	2.8	0.0–5.4	0.0–27.2
Hearing acuity -HV2048 left (Db)	548	17.0	9.9	5.0–36.0	0.0–100.0
Hearing loss left 2048 Hz [%]	548	2.8	4.0	0.2–9.8	0.0–40.0
Hearing acuity -HV2048 right (Db)	549	18.3	10.1	6.0–37.5	0.0–88.0
Hearing loss right 2048 Hz [%]	549	3.2	4.5	0.2–11.4	0.0–39.2
Hearing acuity -HV4096 left (Db)	548	18.0	13.6	3.0–44.0	0.0–100.0
Hearing loss left 4096 Hz [%]	548	1.4	2.3	0.1–6.4	0.0–15.0
Hearing acuity -HV4096 right (Db)	549	18.5	13.4	3.0–45.5	0.0–100.0
Hearing loss right 4096 Hz [%]	549	1.4	2.4	0.1–6.4	0.0–15.0
Hearing acuity -HV512 left (Db)	551	14.9	7.5	5.0–26.0	0.0–100.0
Hearing acuity -HV512 left (%)	551	1.8	2.7	0.2–4.9	0.0–40.0
Hearing acuity -HV512 right (Db)	551	18.1	7.7	8.0–30.0	0.0–79.0
Hearing acuity -HV512 right (%)	551	2.8	3.0	0.4–7.2	0.0–35.8
Start rate [Hz]	608	6.6	0.9	5.2–8.1	2.0–9.2
Test motivation [Hz]	608	5.9	0.7	4.9–6.9	2.3–8.4
Tapping – basic rate, part 3 (Hz)	608	6.1	0.7	4.8–7.0	0.9–8.4
Psychomotor endurance [Hz]	608	5.9	0.7	5.1–7.3	0.1–8.2
Viseomotor coordination ability (mistakes) [n]	606	13.2	7.0	3.0–26.0	1.0–39.0
Viseomotor coordination ability (time) [sec]	606	29.8	14.3	12.8–57.9	6.5–103.2
Cognitive and mental parameters					
Optical reaction time [msec]	613	277.3	37.4	221.7–339.3	195.0–460.0
Acoustical reaction time [msec]	614	283.4	40.7	217.0–347.3	176.0–475.0
Pursuing reaction time [msec]	615	68.4	27.7	31.0–118.0	19.0–210.0
Verbal reaction time [sec]	612	10.8	1.8	8.2–14.0	7.2–19.9
Cognitive reaction time [sec]	612	12.9	2.3	9.8–16.8	7.9–24.0
Cognitive switching capability [sec]	612	22.7	7.7	14.6–34.6	10.4–113.0
Ability to concentrate (mistakes) [n]	611	1.5	2.0	0.0–6.0	0.0–14.0
Ability to concentrate (time) [sec]	611	121.1	37.9	72.9–188.7	52.1–320.4
Orientation capability [n]	614	50.0	14.2	38.0–71.0	30.0–181.0
Strategic thinking [sec]	614	153.0	67.3	87.4–261.0	64.3–779.9
Memory performance [n]	614	95.2	21.0	85.0–125.3	30.0–313.0
Change over capability [sec]	614	1.05	0.4	0.6–1.6	0.5–6.6
Emotional-social parameters					
Stress disposition [score]	464	27.3	5.3	18.0–35.8	10.0–40.0
Social dominance [score]	464	26.7	5.2	17.0–35.0	9.0–40.0
Social power [score]	464	16.1	4.8	9.0–25.0	6.0–35.0
Stress exposition [score]	464	30.8	5.2	22.0–38.8	12.0–42.0
Physical wellbeing [score]	615	3.4	3.9	0.0–11.2	0.0–24.0
Emotional wellbeing [score]	615	1.7	2.6	0.0–8.0	0.0–14.0
Complaint questionnaire - physical and emotional wellbeing [score]	615	5.1	5.6	0.0–17.0	0.0–32.0
Social activity / leisure [score]	615	52.6	13.7	31.2–74.5	12.0–86.0
Social activity / duties [score]	615	90.3	11.0	70.5–106.3	50.0–118.0
Sense of coherence [score]	346	51.1	7.0	37.0–61.0	29.0–63.0
Age parameters					
Chronological age [years]	617	39.6	14.8	–	20.0–78.7
Bio-functional age (BFA) [years]	315	44.1	8.6	–	20.1–73.7
difference_Age_BFA_Index (chronological – bio-functional age)	316	7.8	8.0	–	−19.4 – 29.6

*Abbreviations*: *BIA* Bioelectrical impedance analysis, *Xc* Reactance (capacitive resistance), *Rc* Resistance (ohmic resistance)

#### Trier inventory for the assessment of chronic stress (TICS)

The TICS is a standardised, validated questionnaire (Cronbach alpha 0.9) [23]. It measures nine aspects of chronic stress, e.g., work overload, social overload, pressure to perform, work discontent, excessive demands at work, lack of social recognition, social tensions, social isolation and chronic worrying. Each of the 57 items is rated on a five-point rating scale assessing how often one has experienced a certain situation within the past 3 months (0 = never, 1 = rarely, 2 = sometimes, 3 = often, 4 = very often). In addition, a standardised screening scale for chronic stress (SSCS) represents a global value for chronic stress exposure within the past 3 months. It covers five out of the nine chronic stress domains. The total score ranges from 0 to 48 points providing three subcategories of perceived chronic stress intensity: below average stress (0 to 11 points), above average stress (12 to 22 points) and extreme stress (> 22 points) [24]. Reference values are provided for three age categories but not for sex.

### Statistical analysis

Statistical analysis was performed by applying SPSS (Statistical Package for the Social Sciences) for Mac (Version 22.0). Data have been analysed descriptively, by t-test and chi-square test, respectively. For correlation analysis, the two-sided Pearson product-moment correlation coefficient was used. Statistical significance was defined as *p* < 0.05.

## Results

### Characteristic of the cohort

#### Age, lifestyle, social and job status

In total, 624 participants (73.7% female, 26.3% male) were recruited, mainly (79.8%) via personal contact. Mean age was 39.5 ± 14.9 years. For BFA assessment, age 35 is an accepted threshold; 313 participants (50.2%) were above age 35. 36.2% of participants reported regular alcohol consumption at least twice a week. 37.2% had at least one drink per day. In contrast, the majority was never-smoker (64.7%) and physically active (till sweating) (75.2%) at least once a week. The majority of participants was living in a partnership (74.8%), almost two thirds (65.4%) were childless, whereas one in four participants had at least two children (27.6%). About half of participants (53.8%) had a degree from university or advanced technical college, respectively. One in five participants (20.2%) had a degree from vocational business school. Most participants (37.7%) worked in the social field with 52.1% being employees and 23.7% students, respectively. Job occupation was at least 50% in more than half (68.6%) of participants, and at least 90% in 41.8%. The monthly gross income was less than 5000 Swiss Francs for 49.4%.

#### Personal and family history

The prevalence of being disease free was 18.8% (*n* = 177). Life threatening events were reported by 11 participants (myocardial infarction *n* = 2; stroke *n* = 4; pulmonary embolism *n* = 5). The prevalence of a positive family history varied and was more than 15% for cancer (40.2%), cardiovascular disease (hypertension 32.4%, myocardial infarction 25.3%, stroke 17.3%, pulmonary embolism 6.3%), metabolic disorder (dyslipidemia 21.6%, diabetes mellitus 15.1%), depression (19.6%) and osteoarthritis (20.5%).

#### Medication

In the BeCS-14 cohort, 356 subjects reported use of any kind of medication (*n* = 617 reports). The major medication groups were dietary supplements (*n* = 177), sexual steroids for contraception (*n* = 128) or menopausal hormone therapy (*n* = 50), psychotropic medication (*n* = 52), analgetics (*n* = 59) and antihypertensives (*n* = 38), respectively.

### Bio-functional status (BFS) and bio-functional age (BFA)

Table 1 presents the single BFS items categorized into the four subdomains 1) physical parameters, 2) sensory physiology and psychomotor parameters, 3) cognitive and mental parameters as well as 4) emotional-social parameters, respectively. In addition, chronological and the calculated BFA are provided for the whole BeCS-14 cohort. The mean difference between chronological and bio-functional age was 7.8 ± 8.0 year equivalents.

### Chronic stress exposure

Table 2 presents the TICS subdomains and the SSCS for the whole BeCS-14 cohort. When comparing to the mean values (T50) of the reference population, the BeCS-14 cohort perceived a similar chronic stress level overall (SSCS) and in the subdomaines work overload, work discontent, lack of social recognition, social tensions and social isolation. In contrast, chronic stress by social overload seemed to be higher whereas chronic stress by pressure to perform, and chronic worrying in particular, were lower within the BeCS-14 cohort.

**Table 2:** Chronic stress exposure assessed by TICS

TICS subscale	Mean (SD)	5th–95th percentile	Range	Maximal score possible	T50 (reference cohort)
Screening scale (SSCS)	13.2 (7.0)	3–26	0–37	48	13
Work overload	11.7 (5.6)	2–22	0–31	32	12.5
Social overload	9.1 (4.3)	2–16	0–23	24	7
Pressure to perform	15.1 (6.0)	5–25	0–33	36	17
Work discontent	8.7 (4.7)	2–17	0–31	32	9
Excessive demands at work	5.1 (3.2)	0–11	0–18	24	4.5
Lack of social recognition	3.9 (2.9)	0–10	0–16	16	4
Social tensions	4.8 (3.3)	0–11	0–22	24	5
Social isolation	5.5 (4.1)	0–13	0–24	24	5
Chronic worrying	4.7 (2.9)	0–10	0–15	16	14

*Abbreviations*: *SD* Standard deviation, *SSCS* Screening scale for chronic stress, *TICS* Trier Inventory for the Assessment of Chronic Stress, *T50* Mean value of reference cohort

In BeCS-14, the mean stress level score assessed by SSCS was 13.2 being slightly higher in women (13.4) compared to men (12.5) (*p* = 0.189) (Table 3). When differentiating between stress intensity categories almost half of the BeCS-14 cohort (45.4%) reported to be exposed to above average stress intensity with an additional 11.1% being exposed to extreme stress (≥ 90th percentile). While there was no sex-specific difference in perceived “above average ”stress exposure, slightly more women (12.1%) than men (8.5%) reported to be exposed to extreme chronic stress (*p* = 0.278).

**Table 3:** Chronic stress intensity within the whole BeCS-14 cohort

	BeCS-14 cohort (*n* = 620)	Females (*n* = 456)	Males (*n* = 164)
Below average stress (score ≤ 11)	43.4% (*n* = 269)	42.5% (*n* = 194)	45.7% (*n* = 75)
Above-average stress (score 12–22)	45.5% (*n* = 282)	45.4% (*n* = 207)	45.7% (n = 75)
Extreme stress (score > 22)	11.1% (*n* = 69)	12.1% (*n* = 55)	8.5% (*n* = 14)

### Correlation between bio-functional status (BFS) and chronic stress exposure (TICS-SSCS)

Overall, of all single BFS items (Table 1) 22 (14 objective, 7 subjective) showed a significant correlation with the TICS-SSCS (Table 4). They were ranked according to their strength of correlation with the sense of coherence (SOC_L9) showing the strongest correlation (rank 1; r = − 0.53, *p* < 0.001). In order to prove that potential confounding factors did not change results a partial correlation analysis was performed exemplarily (Pearson correlation for control variable “emotional wellbeing” = − 0.406, “physical/emotional wellbeing” = -0.432, “physical wellbeing” = − 0.487, “BFA-Index” = − 0.543, “social resonance” = − 0.-491, “social potency” = − 0.532). The correlation size did not change and remained significant for all tests (*p* < 0.001). The handgrip strength of the left hand was the best objective parameter (rank 8, r = − 0.129, *p* = 0.002). Interestingly, none of the BFS category 2 (sensory physiology and psychomotor parameters) or category 3 (cognitive and mental parameters) item list had a significant correlation with SSCS-TICS, respectively. TICS-SSCS and the difference between chronological and bio-functional age were significantly negatively correlated (pearson-correlation − 0.24; p < 0.001). Thus, higher chronic stress exposure was associated with a pro-aging state or less vitality.

**Table 4:** Ranked BFS parameters correlating with TICS-SSCS

Rank	BFS parameter	Correlation to TICS-SSCS	
1	SOC_L9: Sense of Coherence	Pearson-Correlation	−0.53
		Sig. (2-sided)	< 0.001
		N	344
2	Complaint questionnaire: emotional wellbeing	Pearson-Correlation	0.48
		Sig. (2-sided)	< 0.001
		N	611
3	Complaint questionnaire: physical and emotional wellbeing	Pearson-Correlation	0.43
		Sig. (2-sided)	< 0.001
		N	611
4	Complaint questionnaire: physical wellbeing	Pearson-Correlation	0.29
		Sig. (2-sided)	< 0.001
		N	611
5	Giessen-Test: social resonance	Pearson-Correlation	−0.25
		Sig. (2-sided)	< 0.001
		N	460
6	difference between chronological and bio-functional age (diff_Age_BFA_Index)	Pearson-Correlation	−0.24
		Sig. (2-sided)	< 0.001
		N	314
7	Bio-functional age (BFA)	Pearson-Correlation	0.16
		Sig. (2-sided)	0.004
		N	313
8	Giessen-Test: social potency	Pearson-Correlation	0.14
		Sig. (2-sided)	0.002
		N	460
9	Hand grip strength, left [KP]	Pearson-Correlation	−0.13
		Sig. (2-sided)	0.002
		N	594
10	Body cell mass – active cell mass [kg]	Pearson-Correlation	−0.13
		Sig. (2-sided)	0.002
		N	583
11	Body cell mass – body water [kg]	Pearson-Correlation	−0.13
		Sig. (2-sided)	0.002
		N	583
12	Body cell mass – lean body mass [kg]	Pearson-Correlation	−0.13
		Sig. (2-sided)	0.002
		N	583
13	Body height [cm]	Pearson-Correlation	−0.12
		Sig. (2-sided)	0.002
		N	613
14	Giessen-Test: social dominance	Pearson-Correlation	−0.11
		Sig. (2-sided)	0.015
		N	460
15	Performance time for 20 squats [sec]	Pearson-Correlation	0.11
		Sig. (2-sided)	0.006
		N	596
16	Hand grip strength, both hands [KP]	Pearson-Correlation	−0.11
		Sig. (2-sided)	0.006
		N	596
17	Body composition measurement -BIA RC 50 [Ohm]	Pearson-Correlation	0.10
		Sig. (2-sided)	0.014
		N	583
18	Systolic blood pressure [mmHg]	Pearson-Correlation	−0.09
		Sig. (2-sided)	0.022
		N	608
19	Hand grip strength, right [KP]	Pearson-Correlation	−0.09
		Sig. (2-sided)	0.028
		N	598
20	Tapping - basic rate [Hz]	Pearson-Correlation	−0.09
		Sig. (2-sided)	0.035
		N	604
21	Vital capacity [ccm/ml]	Pearson-Correlation	−0.09
		Sig. (2-sided)	0.038
		N	588
22	Body weight [kg]	Pearson-Correlation	−0.09
		Sig. (2-sided)	0.036
		N	613

### Sum score

In a next step, we aimed to construct a sum score out of those BFS items significantly correlating with the TICS-SSCS (Table 4). With the help of a sum score we intended to represent chronic stress exposure by our comprehensive BFS/BFA assessment tool. Ten out of 22 BFS items were chosen of which six were subjective (SOC_L9, complaint questionnaire: physical and emotional wellbeing, social resonance, social potency, social dominance) and four objective items (hand grip strength left and right, performance time, tapping basic rate). The other items could not be integrated in the sum score as they were not independent factors. For example, vital capacity was dependent on body weight, body height and sex, and when adjusting for those variables the significance was no longer evident (women *p* = 0.320, men *p* = 0.601).

As the scoring systems (the units measured) of the selected items were not comparable, we introduced six new categories by the means of visual classification in SPSS ranging from one (“bad ”or a lot of stress) to six (“good ”or little stress). For example, for sense of coherence, social resonance and stress exposition, social dominance, hand grip strength and basic tapping rate, respectively, the highest scores corresponded to the best classification group six. In contrast, for the complaint questionnaire covering emotional and physical wellbeing, social power and performance time, respectively, the lowest scores corresponded to the best classification group six (Table 5). Similar to the correlation analysis performed for the original BFS items and TICS-SSCS (Table 4) the newly introduced categories (1–6) also showed a significant correlation for each of the 10 selected BFS items with TICS-SSCS (Table 6).

**Table 5:** Selected BFS items for constructing a sum score representing chronic stress exposure

	6 = good	5	4	3	2	1 = bad
SOCL_9 [score]	> 60	56–60	51–55	46–50	41–45	< 41
Complaint questionnaire: emotional wellbeing [score]	0	1	2	3	4	> 4
Complaint questionnaire: physical wellbeing [score]	0	1–2	3–4	5–6	7–8	> 8
Social resonance [score]	> 37	35–37	32–34	29–31	26–28	< 26
Social potency [score]	< 11	11–14	15–18	19–22	23–26	> 26
Social dominance [score]	> 32	30–32	27–29	24–26	21–23	< 21
Hand grip strength, left [kp]	> 52	45–52	37–44	29–36	21–28	< 21
Hand grip strength, right [kp]	> 52	45–52	37–44	29–36	21–28	< 21
Performance time [sec]	> 32	29–28	25–28	21–24	17–20	< 17
Tapping – basic rate [Hz]	> 7.0	6.6–7.0	6.1–6.5	5.6–6.0	5.1–5.5	< 5.1

**Table 6:** Correlation between newly classified BFS items (Table 5) and TICS-SSCS. Subjective BFS parameters are presented in bolt and objective BFS parameters in cursive style

	TICS-SSCS
SOC_L9 (classified)	
Pearson-Correlation	−.542^**^
Sig. (2-sided)	.000
N	344
BFB part II (classified)	
Pearson-Correlation	−.484^**^
Sig. (2-sided)	.000
N	611
BFB part I (classified)	
Pearson-Correlation	−.320^**^
Sig. (2-sided)	.000
N	611
Social resonance (classified)	
Pearson-Correlation	−.238^**^
Sig. (2-sided)	.000
N	460
Social potency (classified)	
Pearson-Correlation	−.173^**^
Sig. (2-sided)	.000
N	460
Social dominance (classified)	
Pearson-Correlation	−.106^*^
Sig. (2-sided)	.023
N	460
*Hand grip strenght, left (classified)*	
Pearson-Correlation	−.126^**^
Sig. (2-sided)	.002
N	594
*Hand grip strenght,right (classified)*	
Pearson-Correlation	−.097^*^
Sig. (2-sided)	.018
N	598
*Performance time (classified)*	
Pearson-Correlation	−.104^*^
Sig. (2-sided)	.011
N	596
*Tapping – basic rate (classified)*	
Pearson-correlation	−.095^*^
Sig. (2-sided)	.019
N	604

*Abbreviations*: * = significant at *p* < 0.05, ** = significant at *p* < 0.01

Next, a first sum score was built using all 10 selected BFS items. As its pearson-correlation value was − 0.411 (*p* < 0.0001) and thus inferior to the single BFS item sense of coherence (SOC_L9) (pearson-correlation value − 0.542; *p* < 0.0001), we constructed two more sum scores using either only the subjective BFS items (six out of the selected 10) or only the objective BFS items (four out of the selected 10), respectively. Again, the respective pearson-correlation values were − 0.489 and − 0.145 (p < 0.0001) and thus inferior to the single BFS item sense of coherence (SOC_L9). Finally, we constructed a sum score combining the three most representative items (SOC_L9, complaint questionnaire: physical and emotional wellbeing). Indeed, this sum score was superior to the single BFS item sense of coherence (SOC_L9) (pearson-correlation value 0.564, p < 0.0001).

## Discussion

In the current study, we were able to demonstrate that 1) chronic stress exposure in the BeCS-14 cohort was comparable to that of the TICS reference cohort, 2) the mean difference between chronological age (CA) and bio-functional age (BFA) in BeCS-14 was 7.8 ± 8.0 year equivalents, 3) 22 BFS items (14 objective, 7 subjective) significantly correlated with chronic stress exposure (TICS-SSCS), 4) the sum score composed of SOC_L9 and complaint questionnaire (physical and emotional wellbeing) represented chronic stress exposure best, and 5) higher chronic stress exposure was associated with bio-functional pro-aging (less vitality) in both sexes.

Indeed, the Swiss BeCS-14 cohort had some similarities with the German TICS reference cohort from 2003 in respect to number (BeCS-14 *n* = 624; TICS *n* = 604), mean age (BeCS-14 39.5 ± 14.9 years; TICS 41.1 ± 13.5 years), civil status (living in partnership BeCS-14 74.8%; TICS 78.8%) and education (degree from university or advanced technical college BeCS-14 53.8%; TICS 33.3%) of participants, respectively [23].

In BeCS-14, bio-functional age (BFA) was lower than chronological age (CA). The difference was greater than the expected standard deviations in the age groups between 0.84 and 1.29 year equivalents [19, 22] meaning that on average the cohort was functionally younger than its chronological age. So far, only two cohort studies compared CA to BFA, a prospective pilot study in 36 menopausal women [25] and a cross-sectional study in 371 employees of five occupational groups [26]. In both cohorts, mean CA was comparable to (baseline) BFA. In the first study, BFA was significantly reduced (= improved) by 9.77% after 8 weeks of menopausal hormone therapy [25]. In the second study, the highest discrepancy between CA and BFA was found in the group of male managers being on average 9 years functionally younger while female office workers and nursery school teachers had a slight higher BFA compared to their CA [26]. Mental attitudes and resources towards work, occupational reward and the body fat percentage were found to be relevant predictors for the discrepancy between CA and BFA [26].

Various attempts have been made to assess chronic stress in a subjective or objective manner. Besides the questionnaire TICS [23] there are e.g. the Life Experiences Survey [27], Perceived Stress Scale [28], Stress Reactivity Scale [29] and Perceived Stress Questionnaire [30], respectively, that have all been found to correlate with TICS-SSCS.

Of our complex BFS/BFA assessment tool [8], seven subjective BFS items significantly correlated with chronic stress exposure assessed by TICS-SSCS: (1) sense of coherence, (2) physical and (3) emotional wellbeing ((4) combined items), (5) social resonance, (6) social potency and (7) social dominance. The BFS item sense of coherence (SOC_L9) had the strongest correlation with TICS-SSCS. According to the concept of salutogenesis by Antonovsky, sense of coherence comprises three components, e.g. comprehensibility (belief that things happen in a predictable fashion), manageability (belief that the resources necessary to take care of things are available), and meaningfulness (belief that things in life are a source of satisfaction) [31]. The original questionnaire developed by Antonovsky was adapted to and validated in German [32, 33]. Similar to our study, others also found an association between chronic stress and sense of coherence [34, 35] as well as physical and emotional wellbeing [36–41]. The BFS items social resonance, social potency and social dominance were assessed by the Giessen-Test, a deep-psychosocial personality test [42]. The dimension social resonance reflects an individual’s self-rating of attractiveness and popularity. In our study, there was a significantly negative correlation between social resonance and chronic stress exposure. Thus, a person exposed to chronic stress was more likely to rate him−/herself as being, e.g. unattractive, unpopular, disregarded and criticized at work. This finding supports previous studies showing that negative social resonance was correlated to neuroticism [22] that in turn correlated with chronic stress exposure [40]. So far, the association between chronic stress exposure and social potency as well as social dominance has not been investigated before. The dimension social potency reflects an individual’s readiness to socialize. In our study, there was a significantly positive correlation between social potency and chronic stress exposure. Thus, a person exposed to chronic stress was more likely to rate him−/herself as being, e.g. isolated, conscious in heterosexual contacts and little competitive. The dimension social dominance reflects an individual’s self-awareness of being dominant or submissive. In our study, there was a significantly negative correlation between social dominance and chronic stress exposure. Thus, a person exposed to chronic stress was more likely to rate him−/herself as being, e.g. willingly dominant, impatient and involved in conflicts.

However, psychometric assessment of chronic stress is highly subjective. Accordingly, several attempts have been made to measure stress objectively. Objective assessment tools comprise, e.g. cortisol levels in serum, saliva or urine [43]. However, cortisol levels may fluctuate markedly depending on, e.g. the circadian rhythm [44], nicotine [45] and alcohol consumption [46], food intake [47] and exercising [48]. Thus, the high variability of the HPA axis activity makes it difficult to draw conclusions from random single cortisol levels but would require repeated sampling across longer periods of time [43]. In future, another option for chronic stress exposure might be cortisol level measurement in hair. Yet, age- and sex-specific validation of this technique will be mandatory prior to its clinical use. Besides cortisol level measurements, no other objective parameters have been established for chronic stress assessment.

In our study, 14 objective BFS items significantly correlated with chronic stress exposure assessed by TICS-SSCS: (1) systolic blood pressure, handgrip strength ((2) left, (3) right, (4) both hands), (5) vital capacity, (6) body weight and (7) height, (8) body composition ((9) active cell mass, (10) body water and (11) lean body mass), (12) performance time, (13) tapping, and (14) BFA, respectively. However, as most items are not independent variables we focused on handgrip strength, performance and tapping time.

Handgrip strenghth was significantly negatively correlated with chronic stress exposure in our study. So far, only few studies focused on muscle strength and chronic stress [49, 50]. For example, in veterans suffering from posttraumatic stress disorder handgrip strenghth was fatigable more rapidly and force fluctuations increased at a greater rate during low-intensity contractions in comparison to healthy controls [50]. An explanation for the greater muscle fatigability in chronically stressed people might be an imbalance in both, excitatory and inhibitory neuro-signaling pathways during motor tasks, that may lead to impairments in motor performance [50–52]. In contrast, a study in postmenopausal women did not find a correlation between muscle strenghth and perceived stress [49, 53]. However, our finding must be interpreted with caution as the correlation size was small. Cardiovascular performance including performance time was assessed at submaximal stress level to test the individual fitness level and pre-condition for physical endurance performance, respectively. During the test, the subject was requested to perform 20 squats as fast as possible. In our study, there was significantly positive correlation between chronic stress exposure and performance time indicating that chronically stressed idnividuals required more time to perform the task. The tapping test assesses psychomotoric speed by asking the participant to tap with a pen as fast as possible for 2 min. It may be used as a marker for motivation [54]. In our study, chronic stress exposition was significantly negatively correlated with mean tapping frequency indicating that general motivation was lower in stressed than in non-stressed individuals. These observations fit into the central concept of psychosomatic medicine that is that mind and body are integral aspects of all human function and that illness or alterations in physical functioning may be a consequence of stress. As several BFS items were correlated to chronic stress exposure we aimed to construct a sum score in order to represent chronic stress exposure by a single new parameter integrating subjective and also objective parameters. As some BFS items such as body composition, vital capacity, body weight and height and BFA can only be interpreted in the context of age or sex, respectively, we did not include them in our model. Due to the low prevalence of hypertension in BeCS-14 we also chose to exclude blood pressure in our model. We were able to construct a sum score out of three BFS items (SOC_L9, complaint questionnaire: physical and emotional wellbeing) that represented chronic stress exposure best within our complex generic BFS/BFA assessment tool. However, we still need to primarily rely on subjective items.

Chronic stress exposure has been frequently linked to the development of NCD such as cardiovascular diseases [3] and Alzheimer disease [55]. Furthermore, depression and burnout syndrome [24, 56], sleeping disorders [6] chronic pain [7], disturbances of food intake and body weight [57], and immune dysregulation/immunosenescence [58, 59] were found to be more commen in people with high chronic stress exposure. The impact of chronic stress was also reflected on the molecular and cellular level indicating accelerated aging [60–63]. As our complex generic BFS/BFA assessment tool reflects physical, mental-emotional and social functioning as well as chronic stress exposure our observation that higher chronic stress exposure was associated with bio-functional pro-aging (less vitality) perfectly fits to previous studies showing a negative impact of chronic stress exposure on various body cells, organs and systems.

Our study clearly had some limitations. First, recruitement bias cannot be excluded as advertisement for the study was mainly performed in the “medical” environment of the Inselspital Bern. Secondly, we did not randomize participants according to, e.g. age and sex. Thirdly, our complex generic BFS/BFA assessment tool was strictly non-invasive therefore we were not able to adjust our results to e.g., saliva cortisol levels. Lastly, the study design was cross-sectional so we could not rule out intra-personal day-to-day deviations within TICS.

On the other hand, our complex BFS/BFA assessment tool has been shown to be reliable [8].

## Conclusion

We recently fitted our complex generic BFS/BFA assessment tool into a theoretical model incorporating both, the ICF and AHA concept, resulting in a complex AHA assessment diagnostic tool [8] meeting the EIP-AHA requirements for a diagnostic AHA instrument [9]. Chronic stress is known to be one major contributor to develop NCD. We successfully demonstrated that our complex BFS/BFA assessment tool reflects chronic stress exposure by subjective as well as objective BFS items. The sum score out of three subjective BFS items was the best parameter to reflect chronic stress exposure. Our findings support the applicability of the BFS/BFA assessment tool in all areas of a patient-, customer- or need-orientated health care setting, e.g., in health promotion and prevention of NCDs, in therapy and rehabilitation of chronic health conditions and multi-morbidity.

## Abbreviations

BeCS-14: Bern Cohort Study 2014
BFA: Bio-functional age
BFS: Bio-functional status
CA: Chronological age
EIP-AHA: European Innovation Partnership on Active and Healthy Ageing
ICF: International Classification of Functioning, Disability and Health
NCD: Non-communicable disease
SOC_L9: Sense of coherence test
TICS: Trier Inventory for the Assessment of Chronic Stress
TICS-SSCS: Standardised screening scale for chronic stress (part of TICS)
